# Observations on Exostosis

**Published:** 1851-07

**Authors:** D. Vandenburgh


### OBSERVATIONS ON EXOSTOSIS.

BY D. VANDENBURGH.

The disease of the roots of the teeth, commonly called exostosis, is different, in several respects, from exostosis in bones. It is not, like the latter disease, a growth of bone from bone, but a deposition upon the root, of a material similar to the root itself. It does never, like exostosis in bones, present an internal cancellated structure, but is of the same density and appearance throughout. It is evidently a result of inflammation, and is never found in an active state when inflammation does not exist to a considerable extent. It is not confined exclusively to the roots of carious teeth, though these are more liable to its attacks, but is frequently found upon such as, to all appearances, are sound and healthy above the gum. It is in appearance an enlargement of the root, similar to it in color and density; sometimes in form like a rough irregular belt around the body of the root, leaving its apex of the natural size and shape; sometimes a simple tubercle upon one side, or on the apex; and occasionally it appears evenly spread over the whole surface of the root. It sometimes extends around all the roots of a tooth, forming them into one mass; and Prof. Harris has recorded an instance of two teeth united by it.

The origin of exostosis seems to have very little or no direct relation to temperament or constitutional habit, but is dependent entirely upon local causes. Climate, it may be, may have an indirect agency in its prevalence, since frequent and considerable changes of temperature have no slight agency in enhancing the inflammatory diseases to which exostosis seems more directly to owe its origin.

The commencement of this disease is usually upon some root to which the investing membranes have yet at least a partial attachment, and about which a natural process is going on for its ejectment. This consists of a gradual absorption of the alveolar processes about the root, by which it would in time become loosened and entirely separated from the jaw. This process involves a necessity of increased action or irritation in the part, which is ready to become inflammation with the slightest exciting cause. If, as usually happens, there is an inflammatory action in the gum surrounding the root, or teeth adjoining it, these too points of increased action naturally extend toward each other, and, uniting gradually, increase until the periosteum becomes thickened, and closely interwoven with large, sluggish blood-vessels, some of which, like those of the healthy periosteum, ramify into the bony substance of the root, and by assuming a peculiar action, commence a deposit of new bone around it.

The tendency of inflammation in directing itself toward the increased action of the absorbent or the secreting capillaries, is not only very curious, but very difficult of explanation. Nature seems to have given to each part of the body a peculiar power, by which disease is directed toward such a course as most speedily and effectually to terminate itself. If we take, for an illustration, a tooth, worn down so near the nerve that the action of irritants becomes painful to such an extent as to produce inflammation, we shall find this inflammation, most likely, to act, not on the absorbent, but on the secreting vessels, so that, instead of an absorption or destruction of the nerve, we shall have a new deposit of bone, which, by changing the nature of the tissue, guards it against irritation, and cures the disease. But if we take an illustration from inflammation acting upon the jaw or alveolar processes, we shall find it directing itself *here*, toward the increased action of the absorbents, and not the secreting vessels, as in the tooth. This tendency to peculiar results in different organs, is the only explanation we can give why a disease operating upon different parts, though similar in their nature, and in contact with each other, should produce such dissimilar results. It is in accordance with the natural and peculiar tendencies of the parts, that inflammation should act upon the secretory vessels of the tooth, and the absorbents of the jaw, so that in the occurrence of exostosis, the peculiarity which occasions the new action and deposit of bone, is not so much in the disease itself, as in the tooth which it affects.

Inflammation resulting in exostosis, may arise from diseased gums singly, and sometimes, though not usually, it is communicated from the pulp, through the orifice of the root. Once established, it is fully capable of extending itself, and embracing adjoining teeth, whether previously diseased or healthy. While the periosteum is highly inflamed, and the exostosis is increasing, the root within presents a natural appearance, but the tumor is usually a little whiter, more nearly resembling the tooth bone of the crown. But the exostosis and the root within it, is sometimes of a partially transparent and gelatinous appearance, and softer than healthy tooth-bone or ordinary exostosis. This appearance, however, seems never to occur during the active stage of the disease, but only where, by absorption, the alveolar processes around the root have been removed—or from some other cause the inflammation has partially subsided, thus arresting the deposition of bone, and leaving the root as a foreign substance to be acted upon by the absorbants, which remove a part of the calcareous matter, and thus produce the softness and semi-transparency.

It is not always easy to determine the existence of exostosis from the external appearance of the parts. There are usually, however, some peculiar symptoms attending it. Teeth affected by it are sensitive to pressure, particularly if it be quick or rapid, and the pain, though not sharp, seems to be deeply seated, and felt over a large surface, and is often followed by acute neuralgic twinges shooting along the nerves of the face and head. When the exostosis is large, and situated about the body of the root, an unnatural fullness and breadth of alveolus is observable; but should the disease happen to be upon the point of a long root, it might increase to a considerable size, without giving any such indication of its existence. In every stage of the disease, there is always more or less inflammation in the alveolus and gum surrounding it, though these are not always morbidly sensitive or painful. In fact, the disease often progresses to a considerable extent, without giving any painful indications to the patient of its existence. Still it often increases so as materially to interfere with the surrounding parts, and occasions a dull, constant, pressure-like pain, often expanding into head-ache and neuralgia.

The nerves of such teeth as have lost their crowns by decay, and are affected with this disease, are usually dead before its commencement, but I have never found the nerve canal closed at the point of the root, however thick the exostosis may have accumulated. Where it has extended to teeth whose nerves are alive, it may become the cause of the most afflicting tooth-ache, particularly if their crowns be sound, or their nerves unexposed—for the inflammation, following the periosteum of the root, reflected into the internal canal, may extend to the pulp, which, confined within the unyielding limit of its walls, becomes the seat of the most severe pain, ultimately resulting in the death of the nerve. Only under these circumstances is the inflammation consequent upon exostosis, likely to result in alveolar abscess; but when this occurs after the death of the nerve, and the impossibility of the consequent purulent matter escaping by any other means, an abscess is necessarily formed, which sometimes remains permanently open, and constantly discharging matter; in which case it acts as a palliative to the inflammation about the root, reducing its intensity, and arresting the deposit of bone. Should such a tooth be extracted some time after the formation of the abscess, the exostosis and root will be found in the soft, semi-transparent state previously alluded to.

Though exostosis upon a dead root seldom, if ever results in abscess, an abscess, after subsiding, often leaves a chronic inflammation, which may result in the commencement of exostosis.

Exostosis, when existing to any considerable extent, almost invariably produces neuralgic diseases, often of the most severe character, and by no means necessarily confined to the face and head, but traversing, apparently at their choice and will, every part of the system, and yielding to no remedies so long as the exciting cause remains. The removal of a tooth which is the cause of neuralgia, may bring on a paroxysm of the most alarming nature, and often of considerable duration, but once subsided, never to be felt again, unless other causes of the same nature remain.

In the treatment of this, as in other inflammatory diseases of the mouth, every irritant, and every tooth affected by it, should be at once removed. So long as any tooth thus affected remains in the mouth, all remedial means will be entirely inefficient, and often, when such is the case, the disease, instead of being cured, seems merely roused from its slumber, and to rage with increased violence.—*Amer. Jour. of Dental Science.*

---

*Filling Teeth after the Lining Membrane has become Exposed.*—Dr. W. W. Codman, of Boston, informed us, while on a visit to that city last summer, that he had been in the habit, for several years, of filling teeth, under certain circumstances, after the lining membrane had become exposed, and with very great success. He also stated that he was of the opinion, from a number of experiments which he had made, that the pulp of the tooth, when the operation is successful, sooner or later ossified. He presented us with several teeth in which this had actually occurred.—*Amer. Jour. of Dental Science.*

